# The conjunction of non-consciously perceived object identity and spatial position can be retained during a visual short-term memory task

**DOI:** 10.3389/fpsyg.2015.01470

**Published:** 2015-09-30

**Authors:** Fredrik Bergström, Johan Eriksson

**Affiliations:** ^1^Umeå Center for Functional Brain Imaging, Umeå UniversityUmeå, Sweden; ^2^Department of Integrative Medical Biology, Physiology Section, Umeå UniversityUmeå, Sweden

**Keywords:** non-conscious, durability, priming, conscious experience, perception, working memory

## Abstract

Although non-consciously perceived information has previously been assumed to be short-lived (< 500 ms), recent findings show that non-consciously perceived information can be maintained for at least 15 s. Such findings can be explained as working memory without a conscious experience of the information to be retained. However, whether or not working memory can operate on non-consciously perceived information remains controversial, and little is known about the nature of such non-conscious visual short-term memory (VSTM). Here we used continuous flash suppression to render stimuli non-conscious, to investigate the properties of non-consciously perceived representations in delayed match-to-sample (DMS) tasks. In Experiment I we used variable delays (5 or 15 s) and found that performance was significantly better than chance and was unaffected by delay duration, thereby replicating previous findings. In Experiment II the DMS task required participants to combine information of spatial position and object identity on a trial-by-trial basis to successfully solve the task. We found that the conjunction of spatial position and object identity was retained, thereby verifying that non-conscious, trial-specific information can be maintained for prospective use. We conclude that our results are consistent with a working memory interpretation, but that more research is needed to verify this interpretation.

## Introduction

Non-consciously perceived information can be processed at all levels of the visual system (Rees et al., 2002; Kouider and Dehaene, 2007), and influence executive functions (Lau and Passingham, 2007; van Gaal et al., 2010). It is less clear for how long non-consciously perceived information can be retained and influence behavior. It was previously assumed that non-consciously perceived information is extremely fleeting, and would cease to be detectable within 500 ms after stimulus offset (Greenwald et al., 1996; Mattler, 2005; Dehaene and Changeux, 2011). However, recent studies have found that non-consciously perceived information is more durable than previously assumed. For example, Reber et al. (2012) found that repeatedly presented masked word-pairs could influence decision-making 1 min later, and that hippocampal BOLD signal change at encoding predicted the outcomes. Bar and Biederman (1998, 1999) found behavioral evidence of non-consciously encoded visual repetition priming effects on naming tasks after 15–20 min, and Gaillard et al. (2007) found electrophysiological repetition effects (but no behavioral effects) 47 min after single presentations of masked words. These studies suggest that non-consciously perceived information can form long-lasting latent neural representations reminiscent of consciously encoded hippocampus-based memory (Cabeza and Nyberg, 2000) and visual repetition priming (Henson, 2003) mechanisms.

However, whether or not non-consciously perceived information can be actively maintained for prospective use after stimulus offset (i.e., working memory) is still unclear. Initial findings suggest that working memory can operate on non-consciously perceived information. Soto et al. (2011) used delayed cue-target orientation discrimination tasks to demonstrate that 1–2 non-consciously presented items can be maintained during a distractor-filled delay of up to 5 s. Dutta et al. (2014) later used fMRI to link performance on the delayed cue-target discrimination task to BOLD signal change in the dorsolateral prefrontal cortex (DLPFC), and transcranial direct current stimulation to causally link DLPFC to performance. Pan et al. (2014) found that when a non-consciously presented face matched an interocularly suppressed face, the latter had prior entry into conscious awareness compared to non-matching items, but only when the face was needed for prospective use. We have previously used an attentional-blink paradigm to demonstrate that non-consciously presented information can be maintained during a distracter-filled delay for up to 15 s, which was associated with BOLD signal change in the prefrontal cortex (Bergström and Eriksson, 2014). Accordingly, it has been suggested that working memory can operate on non-consciously perceived information (Soto and Silvanto, 2014).

Little is known about the properties of this non-conscious visual short-term memory (VSTM) and we here aim to further clarify the nature of non-conscious memory representations. To this end we used continuous flash suppression (CFS; Tsuchiya and Koch, 2005) to render stimuli non-conscious during a delayed match-to-sample (DMS) task. CFS has become popular in studies of non-conscious processes, because CFS can in an efficient and easily controlled manner suppress the conscious experience of stimuli for long periods of time (e.g., up to 3 min; Tsuchiya and Koch, 2005). Such long presentation durations can potentially enable more reliable and durable non-conscious representations, compared to for example masked stimuli. Here we wanted to replicate our previous findings that non-consciously perceived information can be retained for at least 15 s (Bergström and Eriksson, 2014) with CFS, and determine which properties of the non-conscious representations that can be retained and influence DMS performance.

## Experiment I

In Experiment I we used CFS to suppress faces that expressed angry or neutral emotions presented within a spatial quadrant of the visual field, with variable delay durations (5 or 15 s), to investigate if non-consciously perceived visual information can be retained for similar delay durations as with attentional blink (Bergström and Eriksson, 2014) and masking (Soto et al., 2011) manipulations.

### Materials and methods

#### Participants

Nineteen healthy participants were recruited from the Umeå University campus area. All participants had normal or corrected-to-normal vision, right-eye dominance, gave written informed consent, and were paid for participation. Two participants were excluded for systematically giving the same response instead of guessing when stimulus was not experienced, one for having an extremely high DMS accuracy of stimuli that were reportedly not experienced (*d*′ = 2.16, > 3 SD above the group mean), and one where CFS did not consistently suppress the appearance of the target stimuli. Thus, 15 participants (*M* = 23 years, 11 females) were included in the statistical analyses.

#### Stimuli and procedure

**Figure 1:**
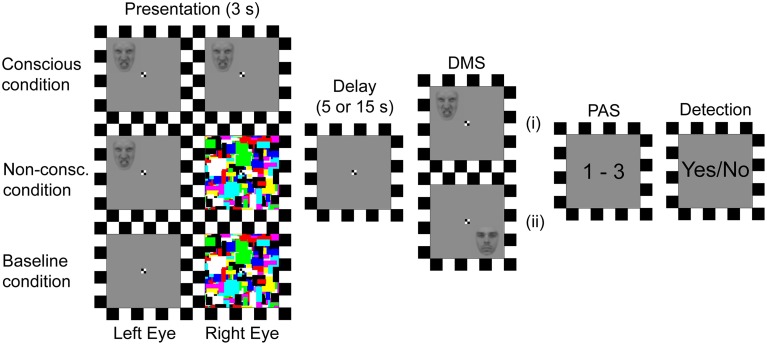
**Trial procedure**. Depending on the presentation condition, two identical stimuli (faces), stimulus and mondrians, or empty background and mondrians were presented to the left and right eye respectively. The spatial position of the stimulus was then to be retained for a 5 or 15 s delay period, until a probe prompted the participants to respond whether or not the probe's position matched the previously presented position. Next, the participants gave an estimate of their perceptual experience of the stimulus. Finally, they responded whether or not a stimulus had been present. DMS, delayed match-to-sample task; PAS, perceptual awareness scale; (i), probe matches presentation; (ii), probe does not match presentation.

The experiment consisted of 270 DMS trials dispersed on three presentation conditions (50 conscious, 170 non-conscious, and 50 “baseline” trials, see below for description), randomly distributed between two delay durations (5 or 15 s) between the stimulus presentation and the DMS response (Figure 1). The stimuli consisted of gray-scaled and Gaussian blurred (1 pixel radius) images of faces (height: 1.6–1.8°, width: 1.3–1.5°, average luminance: 4.3 cd/m^2^) expressing angry or neutral emotions (four faces of each emotion) at 75% opaqueness level (to lower contrast relative to a gray background), and positioned in one of four spatial quadrants. The stimuli were presented on a computer monitor in front of a mirror stereoscope that isolated the visual input from the left side of the monitor to the participants left eye, and vice versa for the right side. The monitor was placed at a length that enabled all visual input to be presented within 6° horizontally and 9.6° vertically. The stimulus to be held in VSTM was presented for 3 s, either to both eyes simultaneously (consciously experienced), or only to the non-dominant (left) eye while colored squares of random composition (mondrians; height: 4.2°, width: 4.2° luminance: ~30 cd/m^2^) where flashed with a frequency of 10 Hz to the dominant eye to suppress the stimulus from conscious experience (Tsuchiya and Koch, 2005). During the baseline trials mondrians were presented to the dominant eye while an empty gray background (height: 4.2°, width: 4.2°, luminance: 8.6 cd/m^2^) was presented to the non-dominant eye. Critically, the visual experience of baseline and non-conscious trials is the same (experiencing only mondrians).

After the delay period a DMS response was prompted by a probe with an identical face, emotion, and spatial position as previously presented (match), or with a different face, emotion, and spatial position (non-match). The participants were instructed to decide if the previously presented stimulus was a spatial match or non-match to the probe (thus, the participants were not instructed to remember face identity or emotional expression). If they had not experienced the target stimulus (i.e., only experienced mondrians) they were instructed to guess on the first alternative that came to mind (match/no match). After the DMS response, they were prompted to estimate the conscious experience of the stimulus on a three-point perceptual awareness scale (PAS; Sandberg et al., 2010). The participants were instructed and trained to use the PAS scale as follows: 1 = no perceptual experience, 2 = vague perceptual experience, and 3 = clear or almost clear perceptual experience of the target stimulus. Lastly, the participants were prompted to make a detection response to determine if a target stimulus had been presented at all (yes or no). If they had not perceptually experienced a stimulus they were to guess per the same instructions as for the DMS task. After participants had received instructions, they performed a practice run of the experiment with the instructor until their behavior was consistent with the instructions, after which the actual experiment started. After the experiment the participants were debriefed and asked about their behavior in relation to the instructions.

#### Statistical analyses

Trials with a DMS response time (RT) of < 250 ms or >M + 3 SD were excluded as outliers prior to any statistical analyses (Ratcliff, 1993). Only trials in the baseline and non-conscious presentation conditions with PAS = 1, and trials in the conscious condition with PAS = 3 were used in the statistical analyses, and will for simplicity hereby be referred to as baseline, non-conscious, and conscious trials. Signal detection theory (d′) was used to calculate performance on the discrimination (DMS) and detection tasks (Macmillan and Creelman, 1991). For DMS d′ the signal was defined as the spatial quadrant where a face appeared. Hits were therefore defined as a match between presentation target and probe together with a “match” response, and false alarms (FAs) as a non-match between presentation target and probe together with a “match” response. For the detection task, hits were defined as the presence of a target stimulus together with a “yes” response, and FA were defined as the absence of a target stimulus (i.e., baseline trials) together with a “yes” response.

### Results

**Figure 2:**
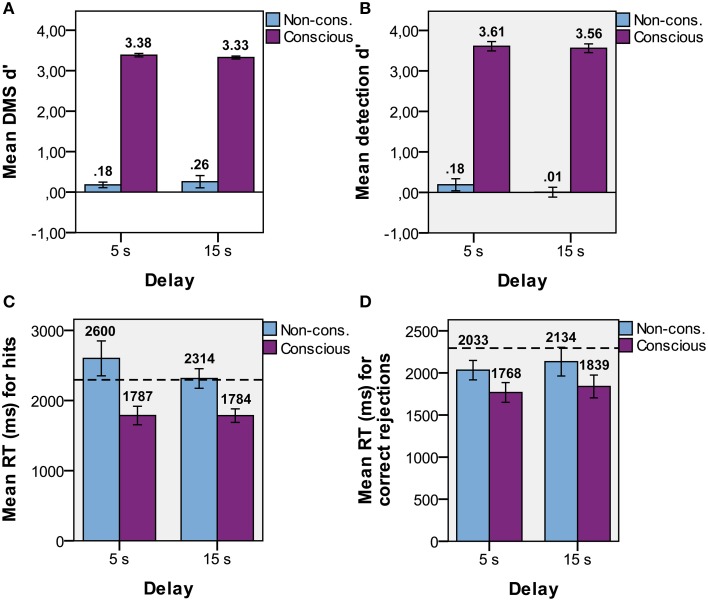
**Task performance**. Mean d′ performance for non-conscious and conscious **(A)** delayed match-to-sample (DMS; discrimination), and **(B)** detection tasks for each delay duration with standard error. Mean DMS response time (RT) for **(C)** hits and **(D)** correct rejections for each delay duration with standard error. Dotted lines represent baseline RT.

The presence of CFS efficiently suppressed the visual input to the non-dominant eye from conscious experience in the non-conscious condition (Table 1). In the following results, all trials with PAS > 1 were removed to ensure no visibility at all of the target stimulus in non-conscious and baseline conditions. A 2 × 2 (visual experience × delay time; Figure 2) repeated measures ANOVA was used to test if DMS performance (d′) changed as a function of (delay) time and/or visibility. There was a main effect of visibility [*F*_(1, 14)_ = 8.3, *p* < 0.001], but not time [*F*_(1, 14)_ = 0.02, *p* = 0.89] and no interaction effect between visibility and time [*F*_(1, 14)_ = 0.94, *p* = 0.35]. Since there was neither a main effect of time nor an interaction effect between time and visibility we proceeded to treat the two time points together. *T*-tests were used to determine if memory performance (Figure 2A) was above chance (i.e., *d*′ > 0), and revealed that non-conscious [*t*_(14)_ = 2.24, *p* = 0.02, one-tailed, *M* = 0.22, *SE* = 0.10, *P*(hits) = 0.47, *P*(FA) = 0.39] and conscious [*t*_(14)_ = 127, *p* < 0.001, one-tailed, *M* = 3.35, *SE* = 0.03, *P*(hits) = 0.95, *P*(FA) = 0.09] DMS d′ was greater than zero. Thus, the DMS results replicated previous research (Bergström and Eriksson, 2014).

**Table 1:** **CFS efficiency**.

**Conditions**	**PAS**		
	**1**	**2**	**3**
Baseline	48	2	0
Non-consc.	128	33	9
Conscious	1	1	48

*Average trial frequency of each presentation condition distributed on reported perceptual awareness scale (PAS) responses per participant*.

For the detection response, a 2 × 2 repeated measures ANOVA showed a main effect of visibility [*F*_(1, 14)_ = 13.4, *p* < 0.001], but not time [*F*_(1, 14)_ = 1.75, *p* = 0.21] and no interaction effect between visibility and time [*F*_(1, 14)_ = 1.27, *p* = 0.28]. Treating both time points together, *t*-tests showed that detection d′ (Figure 2B) was greater than zero for conscious trials [*t*_(14)_ = 33, *p* < 0.001, one-tailed, *M* = 3.58, *SE* = 0.11, *P*(hits) = 0.97, *P*(FA) = 0.11], but not for non-conscious trials [*t*_(14)_ = 0.83, *p* = 0.21, one-tailed, *M* = 0.10, *SE* = 0.12, *P*(hits) = 0.51, *P*(FA) = 0.48]. Thus, participants could not tell whether a target stimulus had been presented or not for the non-conscious trials. The non-conscious DMS d′ and detection d′ were not significantly different from each other [*t*_(14)_ = 1.07, *p* = 0.30].

Repeated measures ANOVAs were used to determine if response time of the DMS task (RT) differed as a function of time and/or visibility for hits and correct rejections (CRs) separately. We hypothesized that RT for both hits and CRs would be faster than baseline if the representation was held in working memory, while only hits would be faster if the representation depended on repetition priming (since there is no stimulus repetitions in CRs). The ANOVA on hits revealed a main effect of visibility [*F*_(1, 14)_ = 21, *p* < 0.001], but not time [*F*_(1, 14)_ = 0.96, *p* = 0.34] and no interaction effect between visibility and time [*F*_(1, 14)_ = 1.02, *p* = 0.33]. Paired *t*-tests on RTs combined over time showed that hits (Figure 2C) were faster than baseline (*M* = 2295, *SE* = 116) for conscious trials [*t*_(14)_ = −4.32, *p* = 0.001, *M* = 1782, *SE* = 89] but not for non-conscious trials [*t*_(14)_ = 1.31, *p* = 0.21, *M* = 2428, *SE* = 147]. The ANOVA on CRs revealed that the main effect of visibility was at trend [*F*_(1, 14)_ = 3.38, *p* = 0.09], but no effect of time [*F*_(1, 14)_ = 1.07, *p* = 0.32] or an interaction effect [*F*_(1, 14)_ = 0.03, *p* = 0.86]. RT on CRs (Figure 2D) was faster than baseline for both conscious [paired *t*-tests; *t*_(14)_ = −3.72, *p* = 0.002, *M* = 1797, *SE* = 117] and non-conscious trials [*t*_(14)_ = −3.69, *p* = 0.002, *M* = 2080, *SE* = 131].

If participants generated a guess of the target directly after the presentation and consciously held that guess in WM during the delay, then the RTs for non-conscious and baseline trials should be equal to the RT for conscious trials. To investigate this issue, we calculated paired *t*-tests for RTs averaged across delay-time, hits, misses, FAs, and CRs. RT for conscious trials (*M* = 1788, *SE* = 93) were faster than RTs for non-conscious [*t*_(14)_ = −3.46, *p* = 0.004, *M* = 2241, *SE* = 131] and baseline [*t*_(14)_ = −4.38, *p* = 0.001] trials, while there was no difference between non-conscious and baseline trials [*t*_(14)_ = −0.97, *p* = 0.35]. These results show that there is a difference between conscious and non-conscious and also baseline trials, presumably because of the extra deliberation time before the guessing response, and indicate that participants had not already guessed the target before the probe appeared. Guessing performance above chance level is thus consistent with non-consciously retained information.

## Experiment II

Experiment I showed that non-consciously perceived information was retained for up to 15 s in a DMS paradigm using CFS to present stimuli non-consciously. However, since the samples were either fully matched or not matched at all in relation to the three information components (spatial position, face identity, and emotion), it is not possible to know which component(s) that were retained in memory. Indeed, spatial attentional effects rather than item-specific memory representations may have driven performance on non-conscious trials. In Experiment II we therefore investigated the content of the information retained by presenting tools at specific spatial positions, and by using probe stimuli that matched in terms of both object and position information (similar to Experiment I), only object, only position, or neither of the information components. It is possible that non-conscious memory mechanisms only retain one of the information components (e.g., the spatial position) despite a conscious task set to retain both. However, if item-specific representations, defined as an arbitrary combination of spatial position and object identity that change from trial to trial, are retained across the delay, the conjunction of spatial- and object information would be preferentially retained over one of the components.

Furthermore, given the uniform effect across delay duration (5 vs. 15 s) in Experiment I, all trials in Experiment II had a 5 s delay period. We chose to use tools as stimuli instead of faces for two reasons: (i) we reasoned that within-category discrimination might be easier for tools because of their distinctly different shapes/features, and (ii) previous research have indicated that tools may be more reliably processed during interocular suppression with CFS (Fang and He, 2005; Almeida et al., 2008).

### Materials and methods

#### Participants

Nineteen healthy participants were recruited from the Umeå University campus area. All participants had normal or corrected-to-normal vision, right-eye dominance, gave written informed consent, and were paid for participation. Two participants were excluded for systematically giving the same response instead of guessing when the stimulus was not experienced, and one where CFS did not consistently suppress the appearance of the target stimuli. Thus, 16 participants (*M* = 25 years, 9 females) were included in the statistical analyses.

#### Stimuli and procedure

**Figure 3:**
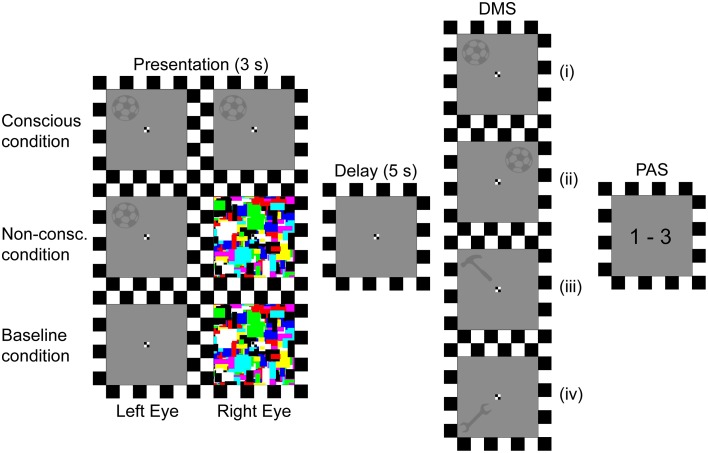
**Trial procedure**. Depending on the presentation condition, two identical stimuli (tools), stimulus and mondrians, or empty background and mondrians were presented to the left and right eye respectively. The stimulus' position and identity was then to be retained for a 5 s delay period, until a probe prompted the participants to respond whether or not the probe's position and identity matched the previously presented position and identity. Finally, they gave an estimate of their perceptual experience of the stimulus. DMS, delayed match-to-sample task; PAS, perceptual awareness scale; (i), Probe identity and position matches presentation; (ii), Probe identity matches presentation; (iii), Probe position matches presentation; (iv), Probe does not match presentation.

The experiment consisted of 396 trials dispersed on three presentation conditions (117 conscious, 222 non-conscious, and 57 baseline trials). The procedure for Experiment II (Figure 3) was identical to that of Experiment I in all aspects except for the following alterations. Firstly, the delay duration between stimuli presentation and response was set to 5 s. Secondly, the detection response was excluded. Thirdly, the stimuli material used was changed from faces to six different gray silhouettes of tools (height: 1.7°, width: 1.7°, Gaussian blur: 1 pixel radius, luminance: 8.4 cd/m^2^). The participants were instructed to remember both the tool and its spatial position. For it to be a “match,” the probe stimulus had to be the same tool and be in the same spatial position (full match). If the probe contained the same tool at a different spatial position (object match), different tool at the same spatial position (spatial match), or different tool at a different spatial position (non-match), it should be answered with a “no match” response.

#### Statistical analyses

Outliers and inclusion criteria for statistical analyses were identical to Experiment I. For DMS d′ the signal was defined as the object identity and its spatial position. Hits were therefore defined as a (position and identity) match between sample and probe together with a “match” response, and FAs as a non-match (which includes cases where only position, only identity, or neither was a match) between sample and probe together with a “match” response.

### Results

**Figure 4:**
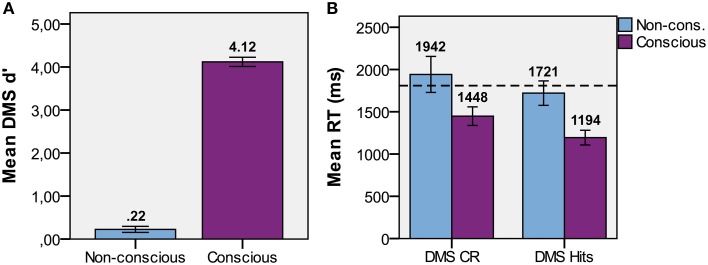
**Task performance**. **(A)** Mean d′ performance for non-conscious and conscious delayed match-to-sample (DMS; discrimination) tasks. **(B)** Mean DMS response time (RT) for hits and correct rejections with standard error, and dotted lines that represents baseline RT.

The presence of CFS efficiently suppressed the visual input to the non-dominant eye from conscious experience in the non-conscious condition (Table 2). In the following results, all trials with PAS > 1 were removed to ensure no visibility at all of the target stimulus in non-conscious and baseline conditions. *T*-tests were used to determine if memory performance was above chance. DMS d′ (Figure 4A) was greater than zero for non-conscious trials [*t*_(15)_ = 3.17, *p* = 0.003, one-tailed, *M* = 0.22, *SE* = 0.07, *P*(hits) = 0.56, *P*(FA) = 0.48] and conscious trials [*t*_(15)_ = 38, *p* < 0.001, one-tailed, *M* = 4.12, *SE* = 0.11, *P*(hits) = 0.97, *P*(FA) = 0.03].

**Table 2:** **CFS efficiency**.

**Conditions**	**PAS**		
	**1**	**2**	**3**
Baseline	53	3	0
Non-consc.	194	26	2
Conscious	3	2	112

*Average trial frequency of each presentation condition distributed on reported perceptual awareness scale (PAS) responses per participant*.

**Figure 5:**
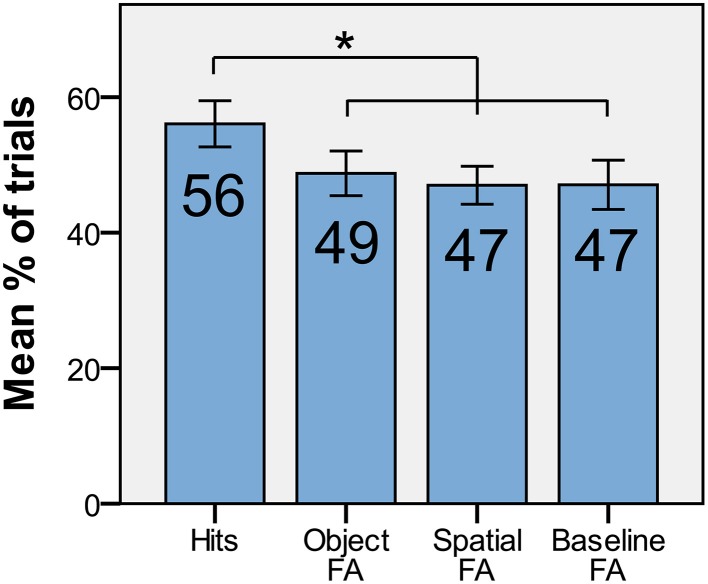
**The average proportion of hits, object-FA, spatial-FA, and baseline-FA with standard error for non-conscious trials**. ^*^p < 0.05, one-tailed.

To examine the relative contributions of spatial position, object identity, and the conjunction of both in driving the non-conscious DMS d′ effect, we looked at the proportion of trials within each of the categories full match, object match, spatial match, and non-match, where participants responded “match.” In short, we compared hit rate (full match) and the false alarm rates when only object identity matched (object FA), only spatial position matched (spatial FA), and when neither matched (baseline FA; Figure 5). A repeated-measures ANOVA showed that there was a difference [*F*_(3, 15)_ = 3.57, *p* = 0.02] among hits/FAs. The planned paired *t*-tests (corrected for three comparisons with the Holm-Bonferroni procedure) revealed that the hit rate for non-conscious trials were greater than baseline FA [*t*_(15)_ = 2.29, *p* = 0.018, one-tailed], object FA [*t*_(15)_ = 2.67, *p* = 0.009, one-tailed], and spatial FA [*t*_(15)_ = 3.12, *p* = 0.004, one-tailed]. There was no difference among FA rates [repeated-measures ANOVA, *F*_(2, 15)_ = 0.19, *p* = 0.83]. These results confirm that the conjunction of spatial position and object identity was retained throughout the delay.

Paired *t*-test comparisons of conscious DMS reaction time (RT; ms; Figure 4B) demonstrated that hits [*t*_(15)_ = 4.22, *p* = 0.001, *M* = 1194, *SE* = 87] and CRs [*t*_(15)_ = 2.79, *p* = 0.014, *M* = 1448, *SE* = 110] were faster than baseline (*M* = 1809, *SE* = 180). However, the paired *t*-test comparisons on non-conscious DMS RT for hits [*t*_(15)_ = −1.09, *p* = 0.29, *M* = 1721, *SE* = 145] and CRs [*t*_(15)_ = 1.83, *p* = 0.09, *M* = 1942, *SE* = 213] were not faster than baseline. Indeed, the CRs RT was at trend with regard to being slower than baseline RT.

Paired *t*-tests of RTs (aggregating hits, misses, FAs, and CRs) showed that RTs of conscious trials (*M* = 1342, *SE* = 99) were faster than RTs of non-conscious [*t*_(15)_ = −3.33, *p* = 0.005, *M* = 1841, *SE* = 187] and baseline [*t*_(15)_ = −3.44, *p* = 0.004] trials, while there was no difference between non-conscious and baseline trials [*t*_(15)_ = 0.70, *p* = 0.50]. These results are consistent with Experiment I in showing that participants had not already decided what to guess before the probe appeared.

## Discussion

In Experiment I, we found that non-consciously perceived visuospatial information can be maintained and influence behavior 5–15 s after stimulus offset, thereby replicating recent findings (Bergström and Eriksson, 2014). Although the participants were instructed to only remember the spatial position, it is possible that face identity and/or emotion information also contributed to performance. For CFS, some have found more reliable processing in the dorsal visual pathway compared to the ventral pathway (Fang and He, 2005; Almeida et al., 2008), but emotional faces (Tsuchiya et al., 2009; Faivre et al., 2012) have also shown to be processed to some extent (see Yang et al., 2014, for a review of CFS research).

In Experiment II, we specifically investigated the content of the non-conscious VSTM representations. We found that spatial position and object identity could be arbitrarily bound on a trial-by-trial basis, and retained for prospective use after 5 s. The specificity of the retained information (i.e., the conjunction of spatial position and object identity rather than one of the components) was further substantiated by the fact that the average proportion of hits was significantly greater than baseline FA, whereas spatial FA and object FA were not. However, in ongoing experiments in our lab we have noted that spatial information may be driving behavioral performance in similar tasks, and we therefore do not exclude the possibility that specific information components may dominate regulation of behavior in different experiments. For example, it is possible that the conscious task set of the participants can bias the non-conscious short-term retention toward a specific content.

Although our current finding that the conjunction of spatial position and object identity can be retained is consistent with previous research on working memory (Jiang et al., 2000; Olson, 2005; Wood, 2011), it does not rule out the influence of other memory mechanisms. However, the use of only six tools and four spatial positions that were reused over the course of the experiment provided proactive interference from previous trials, which makes short-lived unconsolidated long-term memory mechanisms an unlikely explanation for the non-conscious DMS d′ performance (Endress and Potter, 2014). For the same reason, non-consciously encoded hippocampus-based long-term memory (Degonda et al., 2005; Reber et al., 2012; Duss et al., 2014), where each trial is encoded as a specific episodic representation (i.e., a specific temporal context paired with a specific stimulus content), also seems unlikely. Furthermore, the non-conscious retention of information cannot be explained by residual activity in rod and cone receptors (i.e., iconic memory) since such activity tend to subside within 1 s of stimulus offset (Coltheart, 1980; Sligte et al., 2008).

It has recently been suggested that, contrary to common belief, visual object recognition may be position dependent (Kravitz et al., 2008). Kravitz et al. (2010) found decreased object priming performance with changes in spatial position, and a weaker ability to differentiate between object identity (based on BOLD signal change in high-level object-selective cortex) across positions compared to within positions. They therefore argued that high-level object representations are position dependent. If object representations indeed are position dependent and thereby automatically processed together during visual object recogniton, then it is reasonable to assume that this also is the case in lower-level visual memories (e.g., repetition priming), and not unique to higher-level visual memories (e.g., working memory). If true, the arbitrary, trial-specific matching of spatial position and object identity does not by itself exclude an influence of repetition priming.

The DMS task together with the arbitrary binding of spatial position and a limited set of objects would effectively minimize any automatic stimuli-response mapping, and thereby minimize potential priming effects. However, masked priming effects can remain without stimulus-response mapping (Van den Bussche et al., 2009). Although non-consciously encoded priming has been assumed to be short-lived (< 500 ms; Greenwald et al., 1996; Mattler, 2005; Dehaene and Changeux, 2011), there are cases of long-lasting effects (Bar and Biederman, 1998, 1999). The discrepancy between short- and long-lasting non-consciously encoded priming effects might partly be explained by a focus on semantic rather than repetition priming when drawing conclusions about non-conscious priming overall. For example, it could be that non-consciously encoded semantic priming (≤ 100 ms; Greenwald et al., 1996; Draine and Greenwald, 1998) is less durable than non-consciously encoded repetition priming (15 and 20 min; Bar and Biederman, 1998, 1999). Indeed, there is a similar difference in longevity between consciously encoded semantic and repetition priming (Henson, 2003). It would therefore be prudent to assume, a priori, that non-consciously encoded visual repetition priming effects might last for a few seconds and possibly affect performance in a DMS task.

We hypothesized that RTs would be faster than baseline for hits and CRs if the information was held in working memory, but only for hits if the facilitation was caused by repetition priming, since there were no stimuli repetitions to be facilitated during CRs. The RTs for conscious trials in both experiments confirmed our hypothesis regarding working memory, but the RTs for non-conscious trials were variable. Consistent with working memory, the first experiment showed faster RTs for CRs compared to baseline, but this was not true for the second experiment that instead showed a trend to the opposite. Furthermore, non-conscious hits were not faster than baseline in any of the experiments, which is inconsistent with repetition priming effects. The absence of repetition priming effects despite significant discrimination performance on the DMS task is in line with our previous findings (Bergström and Eriksson, 2014), the assumption that repetition priming is less sensitive than recognition memory (Berry et al., 2006), and studies showing that priming has a negligible effect on recognition tasks (Poldrack and Logan, 1997; Conroy et al., 2005). Taken together, the non-conscious RTs did not show convincing support for working memory *per se*, but did not support repetition priming as a likely explanation either.

A likely strategy during trials with a consciously seen target is to verbalize the information, which is consistent with debriefing statements from the participants. Relatedly, a possible objection to the interpretation that DMS performance on trials with non-conscious targets reflects non-conscious working memory is that participants might have verbalized a conscious representation (e.g., “hammer in upper right quadrant”) by guesswork based on non-conscious perception of the target, and then consciously maintained the guess until probed. Such verbalization should generate similar RTs for conscious and non-conscious trials. However, control analyses showed that the RT for non-conscious trials were slower than for conscious trials, suggesting that participants did not use verbalization during non-conscious trials. This is also what the participants reported during post-experiment debriefing. Nevertheless, in principle, such response-time differences could be caused by increased uncertainty rather than strategy differences. Future research may clarify this issue.

There are several accepted approaches for defining the presence/absence of conscious experience. We have here used a subjective measure (the PAS). Subjective measures risk not being completely exhaustive (Reingold and Merikle, 1988) of conscious experiences, and might therefore overestimate non-conscious effects. On the other hand, objective thresholds have been criticized for not exclusively (Reingold and Merikle, 1988) measuring conscious experience, and thereby underestimating non-conscious effects. Indeed, it has been argued that task performance can be an unreliable measure of conscious experience (Lau, 2008). There is currently no consensus on how to most exhaustively and exclusively measure the absence/presence of conscious experience during perception (Boly et al., 2013). However, Sandberg and colleagues (Sandberg et al., 2010, 2014) have shown the PAS to be more exhaustive (and thus more conservative) than other subjective measures such as confidence ratings (Cheesman and Merikle, 1986), as well as objective measures like post-decision wagering (Persaud et al., 2007) and exclusion tasks (Debner and Jacoby, 1994). Nevertheless, caveats regarding subjective measures should be considered in relation to the current results.

In conclusion, we found that non-consciously perceived visuospatial information could be retained for prospective use at least 15 s after stimuli offset, and that object identity and spatial position could be arbitrarily bound and retained for prospective use with a fidelity high enough to enable within-category discrimination after 5 s. Our findings are consistent with the notion of non-conscious working memory, although we cannot, based on the current experiments, completely rule out other memory mechanisms.

### Conflict of interest statement

The authors declare that the research was conducted in the absence of any commercial or financial relationships that could be construed as a potential conflict of interest.
